# Low fasting plasma insulin is associated with atrial fibrillation in men from a cohort study - the Malmö preventive project

**DOI:** 10.1186/1471-2261-14-107

**Published:** 2014-08-24

**Authors:** Linda SB Johnson, Tord Juhlin, Gunnar Engström, Peter M Nilsson

**Affiliations:** 1 Department of Clinical Sciences, Lund University, Skåne University Hospital, Inga-Marie Nilssons väg 49, 20502 Malmö, Sweden

**Keywords:** Atrial fibrillation, Fasting plasma insulin, Hypertension, Population

## Abstract

**Background:**

Type 2 diabetes has been associated with increased incidence of atrial fibrillation (AF) and cardiovascular disease. Controversy remains regarding the role of insulin in the epidemiology of AF risk. The aim of the present study was to study the association between fasting plasma insulin (FPI) and incidence of AF, as well as any effect modification by fasting blood glucose (FBG) or 2 h post-load blood glucose and body mass index (BMI).

**Methods:**

The study population consisted of 6052 men and 1014 women followed for an average of 26.2 years. There were 983 cases of incident AF. Analysis was performed using Cox regression and competing risks regression approaches. The population was analysed as a whole, and by subgroups according to glucose levels and BMI.

**Results:**

After adjustment for age, height, weight, systolic blood pressure and smoking there was a significant inverse association between FPI and AF (hazard ratio; HR) for 4th vs. 1st quartile: 0.69 (95% confidence interval (CI): 0.57-0.83, p < 0.0001) in the cohort as a whole. Among men the corresponding values were HR 0.64 (95% CI 0.52-0.78, p < 0.001) and among women HR 1.16 (95% CI 0.69-1.93, p = 0.58); p-value for interaction 0.06. The protective effects of insulin tended to be weaker in subjects with elevated fasting glucose, implying that the relation between FPI and incident AF could be dependent on the status of individual’s glucose metabolism.

**Conclusions:**

High levels of FPI are associated with lower risk of incident AF in a middle-aged population with a long follow-up.

## Background

Atrial fibrillation (AF) is a well-documented public health concern causing substantial mortality and morbidity [1]. Known risk factors include age, male gender, hypertension and heart disease [2]. However, the established risk factors for AF can only account for approximately half of the AF cases in the population [2-4]. Novel approaches to risk factor screening are thus warranted. A recent meta-analysis has shown diabetes to be a risk factor for AF [5], but controversy remains as to the mechanism involved. There is some evidence that lack of glycemic control increases risk [6,7]. Insulin has been shown to be a risk factor for cardiovascular disease [8] and overall mortality [9]. It is, however, not clear whether insulin or insulin resistance are risk factors for AF. In the Framingham cohort no significant association was found between insulin resistance and AF [10] and in the ARIC study no positive relation was found between fasting plasma insulin (FPI) and AF risk [6]. On the contrary, several studies have shown that the metabolic syndrome, wherein insulin resistance is a crucial component, is related to incidence of AF [11-15]. Thus, some controversy remains regarding the role of insulin in the epidemiology of AF.

Insulin exerts an effect on the vasculature through specific endothelial insulin receptors. The relationship between insulin and the endothelium is complex, and has been shown to vary according to whether the subject is insulin resistant or not [16,17]. In the case of normal insulin sensitivity the effect of the endothelium is to lower blood pressure and enhance blood flow [17] through release of nitric oxide (NO). However, in the insulin resistant endothelium the effect appears to be reversed, with insulin causing vasoconstriction instead and subsequent blood pressure elevation [16]. Therefore, as hemodynamic factors are important risk factors for AF the relationship between insulin and AF could vary according to the individual’s degree of insulin sensitivity and glycaemic control.

Since AF is mainly a disease of the elderly [1,2] the relationship between insulin and cardiovascular mortality could introduce bias in a study that uses conventional Cox regression analysis of the relation between insulin and AF; e.g. subjects that die of other causes are no longer at risk of AF. This problem can be addressed by using the competing risks approach described by Fine and Gray [18] plotting the sub-hazard of individual risk factors.

The Malmö Preventive Project (MPP) is a large population-based cohort with a long follow-up time, with participants recruited through invitation of individuals born in pre-specified years and living in the city of Malmö, Sweden [19]. The present study aimed to investigate the relationship between FPI and incident AF in the MPP, using conventional Cox regression as well as competing risks regression approaches. Furthermore, since there could be an interaction between blood glucose or obesity on the one hand and insulin on the other for AF risk we wished to examine whether the effect of FPI might differ across strata of FBG, 2 h BG and body mass index (BMI).

## Methods

### Study population

The MPP cohort was created at the Department of Preventive Medicine at the University Hospital in Malmö in 1974. It has been described in detail elsewhere [20]. Between the years 1974 and 1992 a total of 22,444 men and 10,902 women participated in the screening (70% attendance rate). The aim was to screen a large proportion of the population and provide intervention to high-risk individuals. Middle-aged men and women born in pre-specified years were invited to participate. The health screening included physical examination, blood sampling, spirometry and a self-administered questionnaire. Interventions (lifestyle modification, drug therapy) were offered to nearly 25% of screened individuals, but there was no significant effect in general on cardiovascular mortality and on overall morbidity [19]. Men were screened mostly during 1974–1982 and women mostly during 1977–1992, but for both genders FPI was measured mainly between 1975–1979, which accounts for the smaller sample size for women.

The study population for the present study was derived from a subgroup of the MPP cohort whose blood samples were analysed for FPI (n = 7298). We excluded 11 cases of prevalent AF and 213 cases of prevalent diabetes at baseline, as well as 1 individual with missing data for smoking status and 7 individuals with missing data for SBP. The study finally included 6052 men and 1014 women. In subgroups by FBG levels individuals with prevalent diabetes were included.

### Data collection

Height (m) was measured using a fixed stadiometer; weight (kg) was measured in light indoor clothing using a balance beam scale. Systolic blood pressure (SBP) (mmHg) was measured twice after 10 min of supine rest, using a sphygmomanometer with a modifiable cuff. Blood samples were drawn after overnight fast and analysed at the Department of Clinical Chemistry, Malmö University Hospital. FBG was analysed using the glucose-oxidase method (1974 to 1977) or the hexokinase-oxidase method (1977 to 1992). These two methods give similar results which were therefore used as such without a conversion factor. FPI was analysed by a nonspecific radioimmunoassay (RIA) method [21] with a detection limit at 3 mIU L^−1^ (nmol L^−1^ = mIU L^−1^ × 7.175/1000). The Department of Clinical Chemistry in Malmö, where the RIA method was originally developed was attached to a continuing standardization program. Individuals who reported that they were currently smoking were classified as smokers. An oral glucose tolerance test (OGTT; 30 g glucose in/ m^2^ body surface at the beginning of the study and a fixed dose of 75 g in the later part of the study) was performed after an overnight fast in randomly selected age cohorts within the study population: in total 5476 (90%) men and 896 (88%) women of the study population. Blood samples were drawn immediately before and after 120 min rest post-OGTT. Sedentary life-style was defined as a positive answer to the question *“Are you mostly sedentary in your spare time”*. Alcohol risk use was assessed as two or more positive answers to the Malmö modification of the Michigan Alcohol Screening Test (MM-MAST) [22] and low socioeconomic status was defined as Statistics Sweden socioeconomic index (SEI) group 11–36.

### National registries

End points were retrieved through linkage with the Swedish Hospital Discharge Register, which is administered by The Swedish National Board of Health and Welfare. A recent validation study showed that the diagnosis of AF or atrial flutter had very high case validity in this register [3]. Participants were followed until first episode of AF (diagnosis codes 427D for the 9th revision of International Classification of Diseases, ICD-9, and I48 for the 10th revision, ICD-10), or until censoring by death or emigration from Sweden. Follow-up ended at June 30th 2009. AF and atrial flutter have not been distinguished due to the similarities of these diagnoses [23]. The study complied with the declaration of Helsinki and has been approved by the Regional Ethics Review Board in Lund (number 85/ 2004).

### Statistics

All analyses were carried out using STATA for Windows version 12.1, excepting sample size calculations which were analysed using the cohort power function in EPISHEET by Rothman (available at http://www.epidemiolog.net). Variables with a positively skewed distribution (FPI and FBG) were log-transformed. FPI was analysed as a continuous variable (per log unit) and in quartiles, using Cox regression and competing risks regression. The population was further analysed stratified on gender, BMI group and FBG interval. In order to evaluate the degree of confounding by baseline SBP and smoking status we fitted two models; *Model 1* adjusted for age, sex, height and weight and, *Model 2* further adjusted for SBP, and current smoking status. The inclusion of variables assessing total cholesterol, triglycerides, sedentary lifestyle, alcohol use and low socioeconomic status did not substantially change the results and these factors were therefore omitted from the final model. In order to evaluate whether changes in screening procedures or calendar time at screening influenced the results we tested whether screening year was associated with outcome. Since it was not it was excluded from the final model.

Models testing for interaction between FPI on the one hand and FBG, BMI, SBP, smoking status, gender and age on the other were fitted, and the models with interaction parameters were tested against those without using likelihood ratio test. Except for gender, for which the interaction parameter was borderline significant, there were no significant interaction parameters, nor any significant differences between models with or without interaction parameters as assessed by the likelihood ratio test. A gender stratified analysis was performed, as well as sample size calculations for women.

The proportionality assumption of cox regression was assessed visually using Nelson-Aalen plots. A two-sided p-value of <0.05 was considered significant.

## Results

Mean follow up was 26.2 years (26.2 years for men and 26.9 years for women), median follow up 30.2 years and inter-quartile range 9.0 years. During this time there were 983 cases of incident AF (875 men and 108 women). Men who received a diagnosis of AF did so after a mean follow-up of 23.4 years, and women after a mean follow-up of 22.9 years. Mean age ± standard deviation at baseline (SD) was 47.2 ± 2.4 for men and 48.3 ± 6.5 years for women. Fifty-one percent of men and 36% of women were current smokers at baseline. Mean SBP was 130 mm Hg for men and 122 mmHg for women. Baseline characteristics by quartiles of FPI are reported in Table 1.

**Table 1:** Baseline characteristics by quartiles of fasting plasma insulin

	**Men**				**Women**			
	**Q 1**	**Q2**	**Q3**	**Q4**	**Q 1**	**Q2**	**Q3**	**Q4**
**Number**	1,423	1,718	1,383	1,528	410	119	258	227
**FPI, mmol/ L, range**	1-3	4-8	9-13	14-260	2-3	4-5	6-10	11-62
**Age, years**	47.0(2.5)	47.1(2.4)	47.3(2.6)	47.6(2.1)	47.8(6.1)	47.9(7.1)	48.1(6.9)	49.6(6.4)
**Height, cm**	176.2 (6.7)	176.3 (6.4)	176.6 (6.6)	176.5 (6.7)	163.4 (5.8)	164.2 (5.8)	163.4 (6.0)	163.2 (5.8)
**Weight, kg**	73.6 (10.1)	75.5 (9.9)	77.7(10.8)	82.9 (13.1)	62.0(9.4)	62.1(9.7)	63.3(9.9)	70.5 (15.6)
**BMI, kg/m**^**2**^	23.7 (2.7)	24.3 (2.8)	24.8 (3.0)	26.6 (3.8)	23.2 (3.3)	23.1 (3.5)	23.7 (3.5)	26.4 (5.4)
**FBG, mmol/L**	4.8 (0.49)	4.9 0.53)	4.9 (0.56)	4.9 (0.60)	4.8 (0.49)	4.8 (0.51)	4.8 (0.48)	5.0 (0.48)
**Blood glucose at 2 h OGTT**	5.2 (1.3)	5.3 (1.4)	5.5 (1.4)	5.9 (1.6)	6.0 (1.4)	6.1 (1.4)	6.6 (1.6)	6.6 (1.5)
**SBP, mmHg**	127(16)	128(15)	130(16)	134 (17)	121 (15)	123 (17)	122 (16)	126 (16)
**Current smoking,%**	54.5	52.0	51.2	47.7	36.3	38.7	34.1	37.7

Values are means (standard deviation) unless otherwise stated.

FPI = Fasting Plasma Insulin, FBG = Fasting blood glucose, OGTT = Oral Glucose Tolerance Test, SBP = Systolic Blood pressure, BMI = Body Mass Index.

### Fasting insulin and prediction of AF

There was a statistically significant inverse association between FPI and incident AF after adjustment for Model 1 covariates, (age, gender, height and weight) as well as after further adjustment in Model 2 (for SBP and smoking status), with HR for 4th vs. 1st quartile: 0.64 (95% CI: 0.52-0.78, p < 0.0001). Results of the analysis using Cox regression are presented in Table 2. There was no substantial difference in results using Cox regression or competing risks regression (Table 3). The association between FPI and incidence of AF was found using the variable as continuous as well as divided into quartiles (Q1-Q4), (p for trend <0.0001). HR (4th vs 1st quartile) was 0.60 (95% CI 0.45-0.80) for individuals below median age (47.6 years) and 0.80 (95% CI 0.61-1.03) for those above the median age. There was no significant interaction between insulin and age with respect to incidence of AF (p = 0.75). In a gender-stratified analysis, the association between FPI and AF was present among men, but not among women. Sample size calculations showed that with the current number of included women and the assumption of the same HR comparing the top to the bottom quartile among women as observed among men (0.66) the probability of detection was underpowered at 0.27. The probability of detecting a HR of 0.8 was only 0.12. The interaction parameter between gender and FPI was borderline significant, p = 0.06.

**Table 2:** Hazard ratios for fasting plasma insulin in relation to incidence of AF

		**Model 1**			**Model 2**		
		**HR**	**95% CI**	**P**	**HR**	**95% CI**	**P**
**Both genders**	Ln-Insulin	0.82	0.75-0.90	<.0001	0.81	0.74.0.88	<.0001
	Quartile 1	reference					
	Quartile 2	0.95	0.80-1.11	0.54	0.95	0.80-1.13	0.54
	Quartile 3	0.75	0.62-0.90	0.002	0.74	0.62-0.89	0.002
	Quartile 4	0.71	0.59-0.86	<.0001	0.69	0.57-0.84	<.0001
**Men**	Ln-Insulin	0.81	0.73-0.89	<.0001	0.79	0.72-0.87	<.0001
	Quartile 1	reference					
	Quartile 2	0.90	0.75-1.08	0.28	0.91	0.76-1.08	0.28
	Quartile 3	0.72	0.59-0.88	0.001	0.71	0.58-0.86	0.001
	Quartile 4	0.66	0.54-0.81	<.0001	0.64	0.52-0.78	<.0001
**Women**	Ln-Insulin	0.98	0.74-1.31	0.91	0.97	0.72-1.30	0.86
	Quartile 1	reference					
	Quartile 2	1.24	0.68-2.29	0.48	1.21	0.66-2.22	0.55
	Quartile 3	0.94	0.56-1.57	0.82	0.95	0.57-1.59	0.85
	Quartile 4	1.18	0.71-1.96	0.52	1.16	0.69-1.93	0.58

Based on 7066 individuals (6052 men and 1014 women) and 983 cases. **Model 1** is adjusted for age, sex (where applicable), height and weight. **Model 2** is adjusted for Model 1 covariates + systolic blood pressure and smoking status. HR for Ln-insulin is expressed per 1 log unit.

**Table 3:** Fasting plasma insulin and incidence of AF analysed with competing risks regression considering mortality as a competing event

		**Model 1**			**Model 2**		
		**SHR**	**95% CI**	**P**	**SHR**	**95% CI**	**P**
**Both genders**	Ln-Insulin	0.81	0.74-0.88	<.0001	0.80	0.73-0.88	<.0001
	Quartile 1	reference					
	Quartile 2	0.99	0.83-1.18	.89	0.99	0.83-1.17	.89
	Quartile 3	0.78	0.65-0.94	0.008	0.78	0.64-0.93	0.007
	Quartile 4	0.69	0.57-0.84	<.0001	0.69	0.57-0.83	<.0001
**Men**	Ln-Insulin	0.80	0.73-0.88	<.0001	0.80	0.72-0.87	<.0001
	Quartile 1	reference					
	Quartile 2	0.95	0.79-1.14	0.59	0.95	0.80-1.14	0.60
	Quartile 3	0.75	0.62-0.92	0.005	0.75	0.62-0.92	0.005
	Quartile 4	0.66	0.54-0.81	<.0001	0.65	0.53-0.80	<.0001
**Women**	Ln-Insulin	0.89	0.69-1.15	0.39	0.87	0.68-1.13	0.31
	Quartile 1	reference					
	Quartile 2	1.25	0.68-2.31	0.47	1.22	0.66-2.26	0.52
	Quartile 3	0.94	0.57-1.57	0.82	0.94	0.56-1.57	0.82
	Quartile 4	1.02	0.61-1.70	0.93	0.98	0.59-1.64	0.95

Based on 7066 individuals (6052 men and 1014 women) and 983 cases. **Model 1** is adjusted for age, sex (where applicable), height and weight. **Model 2** is adjusted for Model 1 covariates + systolic blood pressure and smoking status. SHR for ln-Insulin is expressed per log unit. SHR = sub-hazards ratio from a competing risk regression.

### Risk of AF in relation to fasting insulin and categories of glycaemic control

In Table 4 the association between FPI and AF stratified by 2 h glucose values post-OGTT is presented. FPI was associated with a lower risk of AF in all quartiles (Q1-Q4) of 2 h glucose, but only findings in Q1-Q2 were statistically significant. The p-value for interaction between 2 h glucose and FPI was not significant, p = 0.44.

**Table 4:** Hazard ratios per unit increase in ln-fP-Insulin and incidence of AF; subgroups by quartile of 2 h glucose post-OGTT

	**Model 1**					**Model 2**				
	**HR**	**95% CI**	**P**	**N**	**AF events**	**HR**	**95% CI**	**P**	**N**	**AF events**
**Q1 BG = 1.9-4.4**	0.80	0.65-0.99	0.038	1552	219	0.78	0.64-0.97	0.022	1551	219
**Q2 BG = 4.5-5.4**	0.81	0.66-0.98	0.033	1600	219	0.80	0.66-0.97	0.025	1600	219
**Q3 BG = 5.5-6.4**	0.87	0.72-1.05	0.16	1708	235	0.86	0.71-1.04	0.12	1708	235
**Q4 BG ≥6.5**	0.87	0.73-1.05	0.14	1691	238	0.86	0.72-1.03	0.091	1691	238

Cases of prevalent diabetes included for these sub-analyses.

**Model 1** is adjusted for age, sex, height and weight. **Model 2** is adjusted for Model 1 covariates + systolic blood pressure and smoking status. Analysed using Cox regression. BG= Blood Glucose.

We also performed analyses including cases of prevalent diabetes and stratified by FBG, (Additional file 1: Table S1). Among individuals with a FBG at or above 6.5 mmol/L, FPI was associated with a borderline significantly increased incidence of AF with HR per 1 log unit: 2.04 (95% CI: 0.94-4.44, p = 0.072). The interaction parameter between FBG and FPI was not statistically significant (p = 0.95).

### Risk of AF stratified for categories of BMI

Analyses were also performed stratified by BMI categories. The association between insulin and incident AF was strongest among individuals in the third quartile of BMI (BMI Q3: 24.3-26.6 kg/m^2^), with HR 0.62 (95% CI: 0.52-0.75, p < 0.0001). There was no indication of an association between FPI and incident AF in the second (Q2: 22.5-24.2 kg/m^2^), HR 0.99 (95% CI: 0.82-1.20, p = 0.89) and top quartiles (Q4: >26.6 kg/m^2^ ); HR 0.95 (95% CI: 0.85-1.10, p = 0.50). The interaction parameters between BMI and FPI were not significant, p = 0.42. (Additional file 1: Table S1).

In order to address possible confounding by prevalent and incident coronary artery disease (CAD) and congestive heart failure (HF) we performed a subanalysis in which individuals with known CAD or CHF at baseline were excluded, and individuals were censored at the time of a CAD or CHF event. Results were not substantially altered in this subgroup, for men the HR comparing the 4th to the 1st quartile of FPI was 0.67, (95% CI 0.54-0.82, p < 0.0001).

A model adjusting for *Model 2* co-variates as well as total cholesterol and triglycerides was tested, without any substantial change in the effect measure between FPI and incident AF; per log unit of FPI HR 0.81, (95% CI: 0.74-0.89, p < 0.0001).

## Discussion

The presented population-based cohort with a long follow-up of middle-aged individuals found a statistically significant inverse association between FPI and incident AF. This association remained significant after adjusting for known AF risk factors; age, height, weight, systolic blood pressure and smoking status (Tables 2 and 3). [24-26] We also ruled out confounding by total cholesterol, triglycerides, low socioeconomic status, physical inactivity and risk over-use of alcohol before excluding these factors from the final model. There was a borderline significant evidence of an interaction between gender and FPI, and no significant association between FPI and AF was found among women in a gender stratified analysis, but in the context of the relatively small sample size of women further study would be needed to assess whether this was due to lack of statistical power among women or a true lack of association among women.

Following adjustment FPI displayed an inverse linear relationship with incident AF among normoglycemic men. Therefore, we conclude that in the MPP cohort high levels of FPI at baseline confer a decreased risk of incident AF during follow-up in the population as a whole and among normoglycemic men.

The inverse association between insulin and AF was somewhat weaker in subjects with high 2 h glucose post-OGTT in the top quartile, and a positive association was observed in subjects with high fasting blood glucose, which was close to statistical significance. There were no statistically significant interaction parameters between FBG or 2 h glucose post OGTT on the one hand and FPI on the other. These results could thus be due to chance, but it would be interesting to examine whether the association between FPI and AF risk, could be dependent on the degree of the subject’s glycaemic control in a larger sample with a higher proportion of subjects with high glucose values.

The results of the present study differ from the results of the ARIC study, which found no significant association between fasting insulin and incident AF [6]. The mean age of the ARIC study was 57 years; roughly ten years older than subjects of the present study at baseline. The mean BMI in the ARIC study was higher as well, corresponding to the top quartile in the present study, where the inverse association between FPI and AF in our data appeared weakest, and was not statistically significant. Therefore, it could be that the results in the ARIC cohort differed from the present study because that study included a higher proportion of individuals with disturbed glucose metabolism, in whom FPI tended to be associated rather with an increased risk of AF, thus tending to nullify the overall risk of insulin. The same argument holds true for the Framingham study which reported no relationship between insulin resistance and incident AF in subjects with a mean age of 59 years and a mean BMI of 27.4 kg/m^2^[10].

The MPP cohort thus examines the relationship between FPI and incident AF in a younger and healthier cohort as compared to the ARIC and Framingham cohorts. It can therefore be assumed that the MPP includes a larger proportion of individuals in a strictly normoglycemic state as compared to the other studies. This may be of importance since the effects of insulin on the vasculature have been shown to vary according to whether the individual is insulin resistant or not [16].

Insulin acts on the vasculature through specific endothelial insulin receptors. It is well known that the insulin hormone normally has a vasodilator effect [27], and increases skeletal blood flow [28]. This effect is mediated through NO [29] following signaling through the phosphatidylinositol 3-kinase - dependent (PI3K) pathway, and can be inhibited by use of a competitive inhibitor of nitric acid synthase [30]. Further proof of the vasodilator effect of insulin is that insulin sensitizing drugs such as glitazones (thiazolidinediones) have a well-known, albeit small, blood pressure lowering side-effect [31,32]. In insulin resistance, however, PI3K mediated signaling is impaired, resulting in vasoconstriction [33,34].

It is well documented that blood pressure is an important AF risk factor, including high normal blood pressure [35-38]. Furthermore, it has recently also been shown that low insulin secretion is associated with increased incidence of hypertension in studies of families at genetic risk of type 2 diabetes [39]. It is therefore possible that different actions of insulin on the vasculature and hemodynamic regulation, and thereby the blood pressure levels, could contribute to explain the results of the present study. It is also possible that these effects could differ between individuals with impaired or normal insulin sensitivity.

The findings of the present study could be of importance for the understanding of the relation between disturbed glucose metabolism, insulin regulation and sensitivity, and AF risk. To the best of our knowledge the MPP is the first cohort study to examine the relationship between FPI and AF in relation to shifting glycaemic control.

### Strengths and limitations

The high participation rate, large size and long follow-up represent strengths of this study, as well as the high quality and proven validity of the Swedish National Hospital Discharge register [3]. We have controlled for many known risk factors for AF, and ruled out confounding by others. However, we still cannot rule out residual confounding, for example influences by thyroid disease, sleep apnea or valvular heart disease. Given the relatively low mean age of the cohort, any such confounding can be assumed to be small, however, and not on a scale to influence results. We have adjusted for height and weight, but it is possible that another measure of body composition, such as waist circumference, would have accounted for the possible confounding from body composition on the association between FPI and AF better, and that some residual confounding remains.

Cases with AF were retrieved from the Swedish Hospital discharge register. A recent validation study showed that case validity of this diagnosis is very high [3]. This register has been operating in southern Sweden during the entire follow-up period and became nation-wide in 1987. However, many AF patients are treated as outpatients without any hospitalization and these cases have been missed. Prevalent cases were retrieved from the same database. We cannot rule out that some individuals with subclinical and undetected AF at baseline could have erroneously been included in the study, however, given the relatively low mean age of the cohort this number can be assumed to be small.

## Conclusions

The present study found an inverse association between FPI and risk of incident AF during long-term follow-up for the population as a whole and among men. This association is not explained by previously known risk factors for AF. Further study of the glucose-dependent relation between insulin and incident AF risk is warranted.

## Competing interests

The authors declared that they have no competing interests.

## Authors’ contributions

PN and GE conceived of the study. All authors participated in study design. LJ carried out analyses with the participation of GE and drafted the manuscript. All authors contributed equally. All authors revised and approved the final manuscript.

## Pre-publication history

The pre-publication history for this paper can be accessed here:

http://www.biomedcentral.com/1471-2261/14/107/prepub

## Supplementary Material

Additional file 1 **Table S1.** Hazard ratios per unit increase in ln fP-Insulin and incidence of AF analysed with Cox regression; subgroups by fasting blood glucose (FBG). **Table S2.** Hazard Ratios per unit increase in ln fP-Insulin and incidence of AF, stratified by quartile of BMI. **Table S3.** Baseline characteristics, comparison between men and women. Click here for file
