# Supplementary material for: Low fasting plasma insulin is associated with atrial fibrillation in men from a cohort study - the Malmö preventive project
Source: BMC Cardiovasc Disord. 2014 Aug 24;14:107. doi: 10.1186/1471-2261-14-107 (PMC4236524; doi:10.1186/1471-2261-14-107)
Supplement: Additional file 1 — Table S1. Hazard ratios per unit increase in ln fP-Insulin and incidence of AF analysed with Cox regression; subgroups by fasting blood glucose (FBG). Table S2. Hazard Ratios per unit increase in ln fP-Insulin and incidence of AF, stratified by quartile of BMI. Table S3. Baseline characteristics, comparison between men and women. [file 1471-2261-14-107-S1.docx]

**Additional file**

**Table S1.** Hazard ratios per unit increase in ln fP-Insulin and incidence of AF analysed with Cox regression; subgroups by fasting blood glucose (FBG).

|  |  | Model 1 | | | | |  | Model 2 | | | | |
| --- | --- | --- | --- | --- | --- | --- | --- | --- | --- | --- | --- | --- |
|  |  | HR | 95% CI | p | Cases | Events |  | HR | 95%CI | p | Cases | Events |
|  | FBG <5.6 | 0.81 | 0.73-0.89 | <.0001 | 6305 | 868 |  | 0.79 | 0.72-0.88 | <.0001 | 6300 | 867 |
|  | FBG 5.6-6.0 | 0.88 | 0.66-1.16 | 0.36 | 650 | 101 |  | 0.86 | 0.65-1.14 | 0.28 | 648 | 101 |
|  | FBG 6.1-6.4 | 0.80 | 0.49-1.29 | 0.36 | 154 | 24 |  | 0.74 | 0.44-1.24 | 0.26 | 154 | 24 |
|  | FBG ≥6.5 | 2.18 | 1.00-4.74 | 0.049 | 168 | 21 |  | 2.04 | 0.94-4.44 | 0.072 | 168 | 21 |

**Model 1** is adjusted for age, sex, height and weight. **Model 2** is adjusted for model 1 covariates and systolic blood pressure and smoking status. Patients with diabetes are not excluded for this sub-analysis.

**Table S2** Hazard Ratios per unit increase in ln fP-Insulin and incidence of AF, stratified by quartile of BMI.

|  |  | Model 1 | | |  | Model 2 | | |
| --- | --- | --- | --- | --- | --- | --- | --- | --- |
| Quartile | BMI | HR | 95% CI | p-value |  | HR | 95% CI | p-value |
| 1 | 14.3-22.5 | 0.82 | 0.65-1.03 | 0.08 |  | 0.82 | 0.66-1.03 | 0.09 |
| 2 | 22.5-24.2 | 0.99 | 0.82-1.21 | 0.95 |  | 0.99 | 0.82-1.20 | 0.89 |
| 3 | 24.3-26.5 | 0.63 | 0.52-0.76 | <.0001 |  | 0.62 | 0.52-0.75 | <0.0001 |
| 4 | ≥26.6 | 0.99 | 0.86-1.15 | 0.89 |  | 0.95 | 0.82-1.10 | 0.50 |

**Model 1** is adjusted for age and gender. **Model 2** is adjusted for model 1 covariates + systolic blood pressure and smoking status.

**Table S3** Baseline characteristics, comparison between men and women

|  | Men | Women | p-vaule |
| --- | --- | --- | --- |
| Age, years | 47.2 (2.4) | 48.3 (6.5) | <0.0001 |
| BMI, kg/m^2^ | 24.8 (3.3) | 24.0 (4.1) | <0.0001 |
| FBG, mmol/L | 4.9 (0.6) | 4.8 (0.5) | 0.002 |
| Blood glucose at 2h OGTT, mmol/L | 5.5 (1.5) | 6.3 (1.5) | <0.0001 |
| SBP, mmHg | 130 (16) | 123 (16) | <0.0001 |
| Current smoking, % | 51 | 36 | <0.0001 |

All values are mean (Standard Deviation) unless stated otherwise.

BMI= Body Mass Index, FBG= Fasting blood glucose, OGTT= Oral Glucose Tolerance Test, SBP= Systolic Blood Pressure

Normally distributed continuous variables (age, BMI, SBP, Blood Glucose at 120´OGTT) were analysed using t-test, skewed variables (FBG) with Mann-Whitney U-test and categorical variables (smoking status) with Chi^2^-test.
